# Supplementary material for: Genome-scale chromatin binding dynamics of RNA Polymerase II general transcription machinery components
Source: EMBO J. 2024 Apr 2;43(9):1799–821. doi: 10.1038/s44318-024-00089-2 (PMC11066129; doi:10.1038/s44318-024-00089-2)
Supplement: Supplementary file 1 — Appendix [file 44318_2024_89_MOESM1_ESM.pdf]

Appendix

Genome-scale chromatin binding dynamics of RNA Polymerase II general transcription machinery components

Kristyna Kupkova, Savera J. Shetty, Elizabeth A. Hoffman, Stefan Bekiranov, and David T. Auble✉

✉ Correspondence: auble@virginia.edu.

TABLE OF CONTENTS

Supplementary figures 2

Appendix Figure S1- Competition ChIP. 2

Appendix Figure S2 - Residence time PCA. 3

Appendix Figure S3 – Example ChIP signal tracks. 4

Appendix Figure S4 - Heatmap clusters enrichment. 5

Appendix Figure S5 - Comparison of residence times between genes with or without a consensus TATA element in their promoters. 6

Appendix Figure S6 - Comparison of residence times between GAL genes and others. 7

Appendix Figure S7 - Functional enrichment of long-lived and short-lived TFIIF sites. 8

Appendix Figure S8 - Comparison to Rap1 residence times and comparison of TBP and TFIIE residence times to others. 10

Appendix Figure S9 - Growth of competition ChIP strains. 11

Supplementary tables 12

Appendix Table S1 - Quantified Western blots: HA/Myc ratios normalized to 0-1 12

Appendix Table S2 - List of primers 13

Appendix Table S3 - Hill model fits to HA/Myc Western blot data for turnover model 14

Appendix Table S4 - Accurate initial estimates of residence times for turnover model fits: 14

Supplementary references 15

SUPPLEMENTARY FIGURES

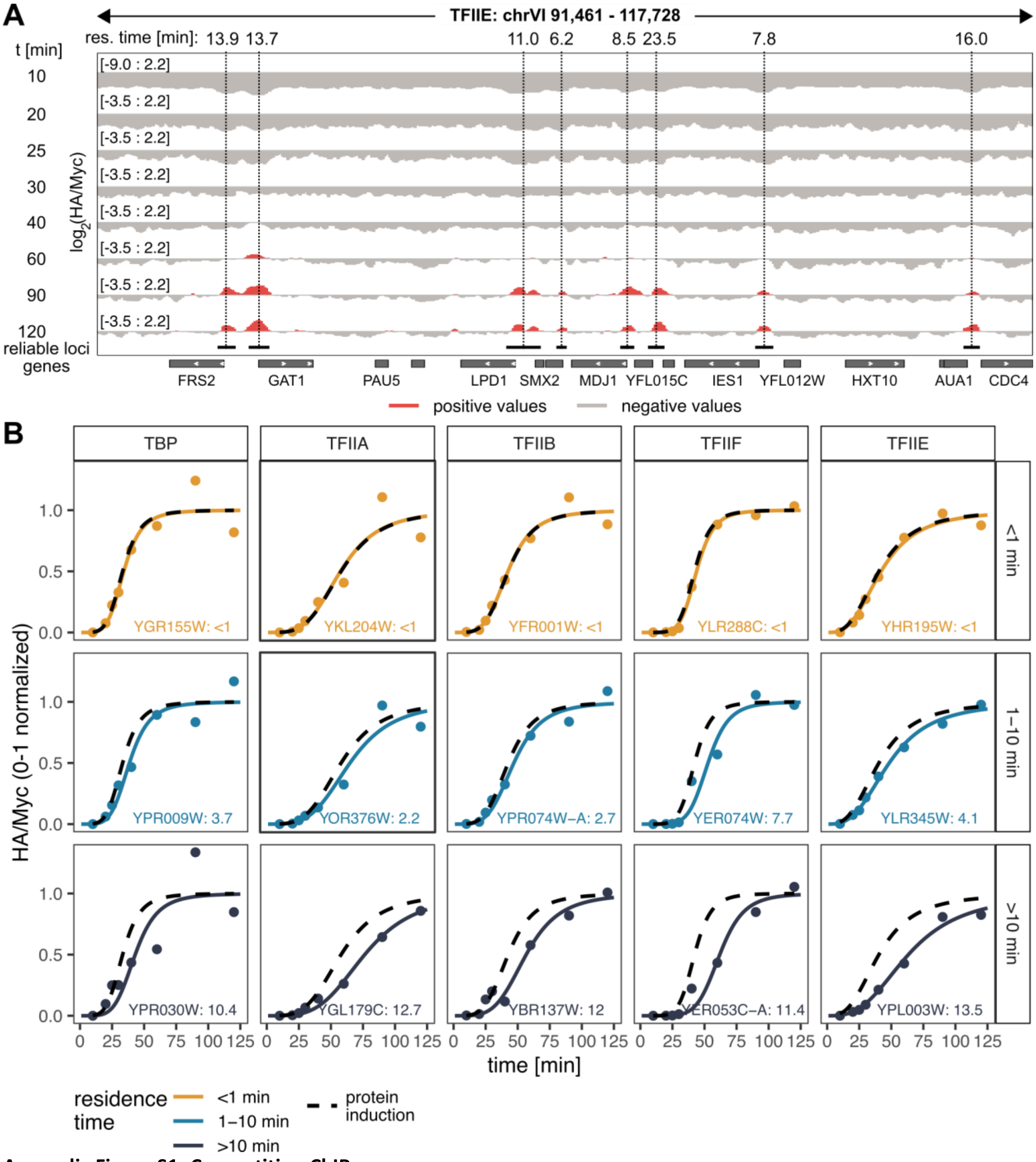

Appendix Figure S1- Competition ChIP.

A Genome browser view showing log_2 transformed ratios of normalized HA and Myc ChIP signals over time.

B Examples of fitted ChIP-seq data for fast (<1 min), moderate (1-10 min) and slow sites (>10 min) for all GTFs examined in this study. Corresponding gene targets are indicated in the bottom right corner of each panel along with the estimated residence times in minutes. The plots show the normalized measurements of HA and Myc ChIP signal ratios fitted with colored curves; the dashed black curves show the induction rate for a given competitor.

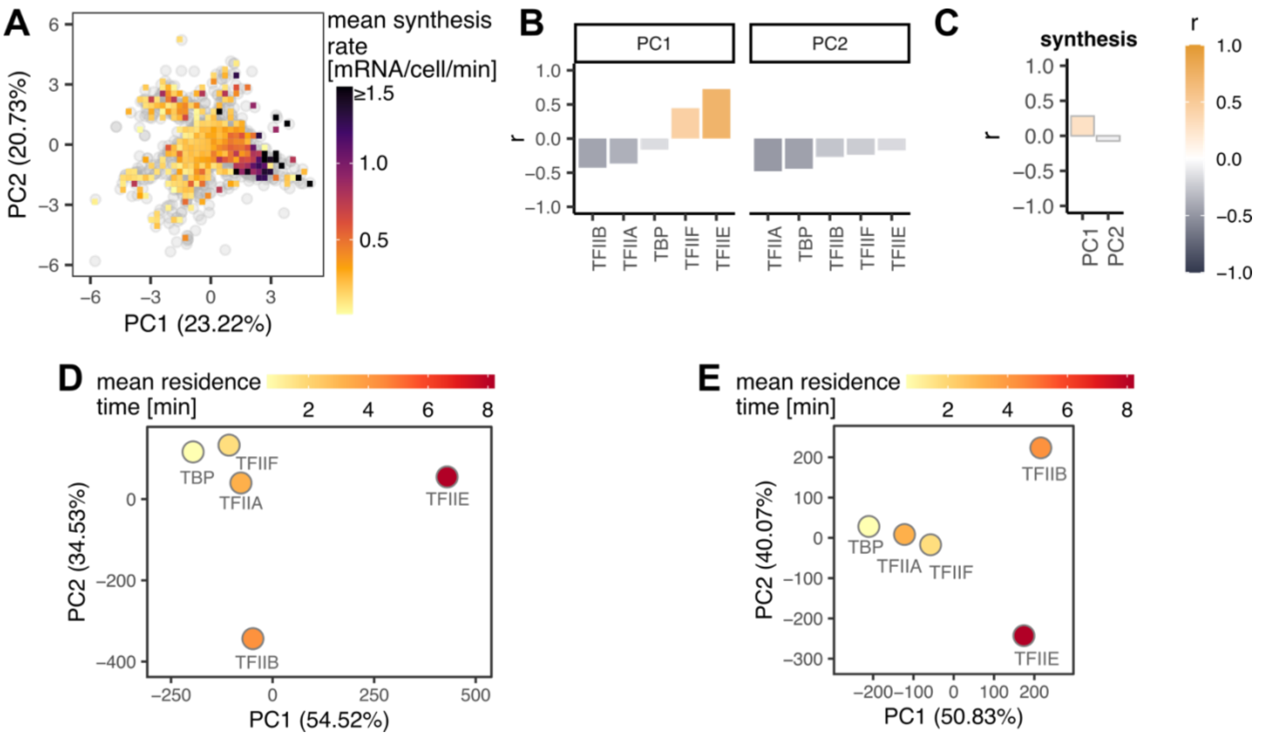

Appendix Figure S2 - Residence time PCA.

A PCA plot separating genes (points) based on GTF residence times. Color coding indicates the mean synthesis rate of the genes falling under a given area. Note that in comparison to Fig 3E, in this figure genes with residence time estimates <1 min were excluded from the analysis.

B Person's correlation coefficients (y-axis) between PCs from (A) and residence times of a given GTF (x-axis).

C Pearson's correlation coefficients (y-axis) between PCs (x-axis) from (A) and synthesis rates.

D PCA plot separating individual GTFs (points) based on their residence times.

E Similar to (D), however, sites with residence time estimates <1 min were excluded from the analysis.

Data information: In (A,D,E) the percentages in the axis labels indicate percentage of variance explained by a given PC.

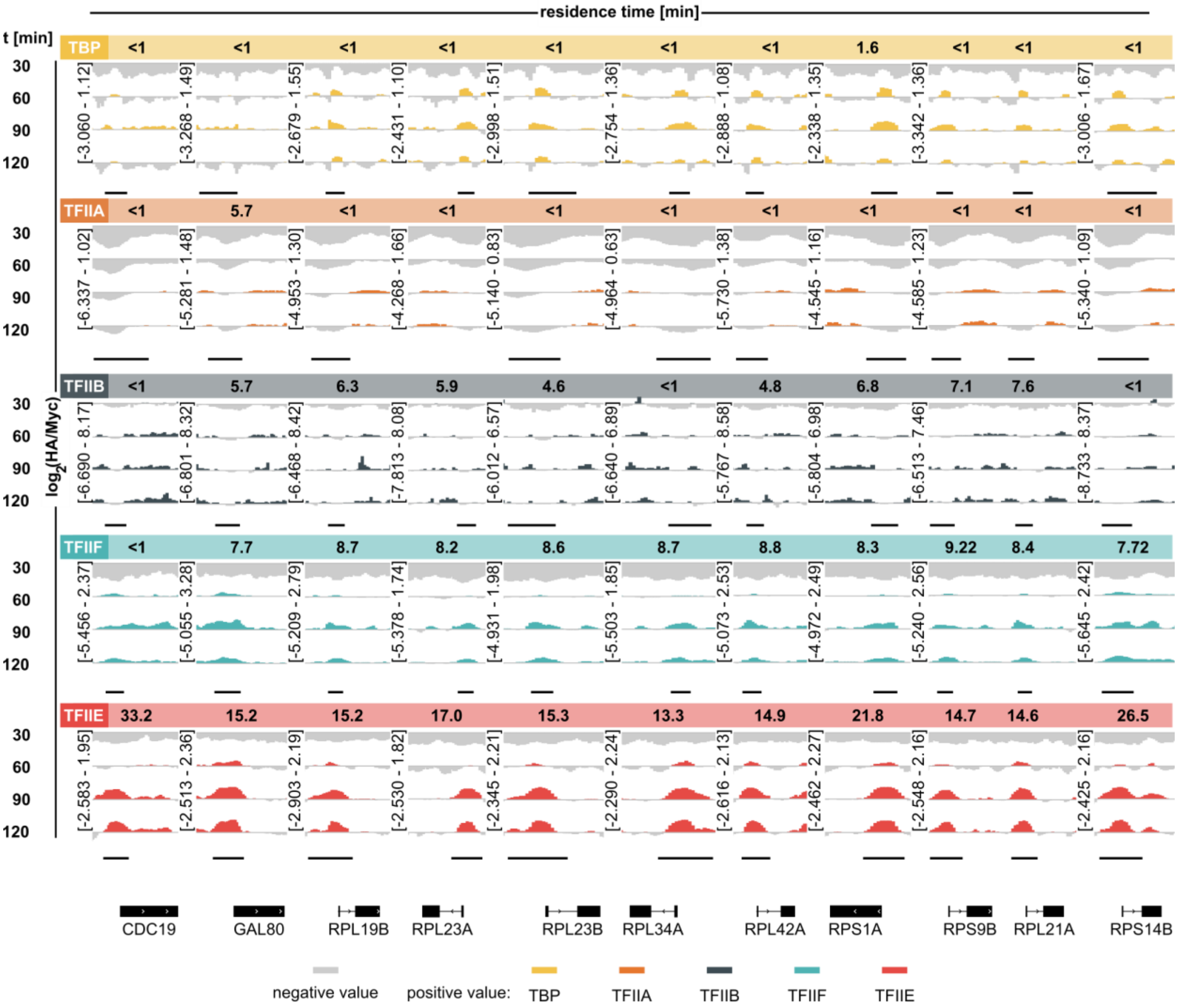

Appendix Figure S3 – Example ChIP signal tracks.

Genome browser views showing log_2 transformed ratios of normalized HA and Myc ChIP signals over time for all GTFs. Shown are example genes from cluster 1 (Fig 4A).

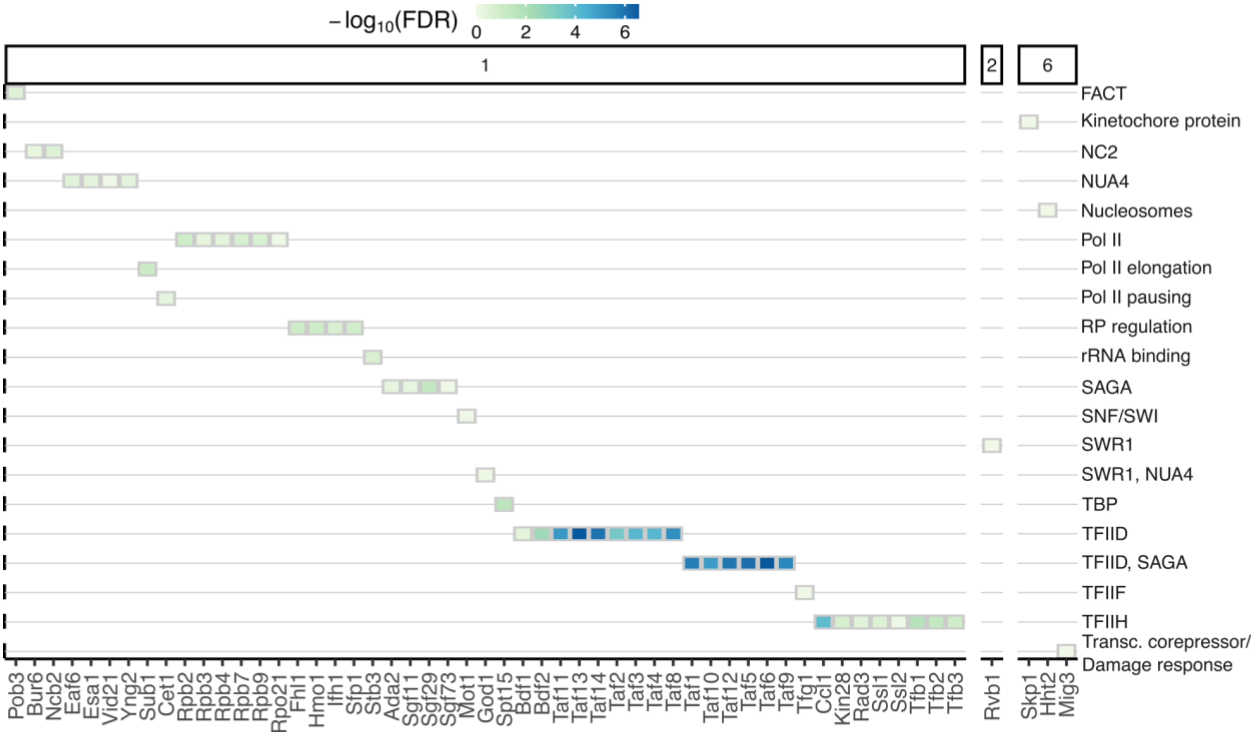

Appendix Figure S4 - Heatmap clusters enrichment.

The figure shows the yeast epigenome database transcription factors enriched (Fisher's exact test) in clusters in Fig 4A classified into manually curated functional groups. Compared to Fig 4D, subunits of GTFs and Pol II are included. The numbers on the top of the plot indicate the cluster number from Fig 4A.

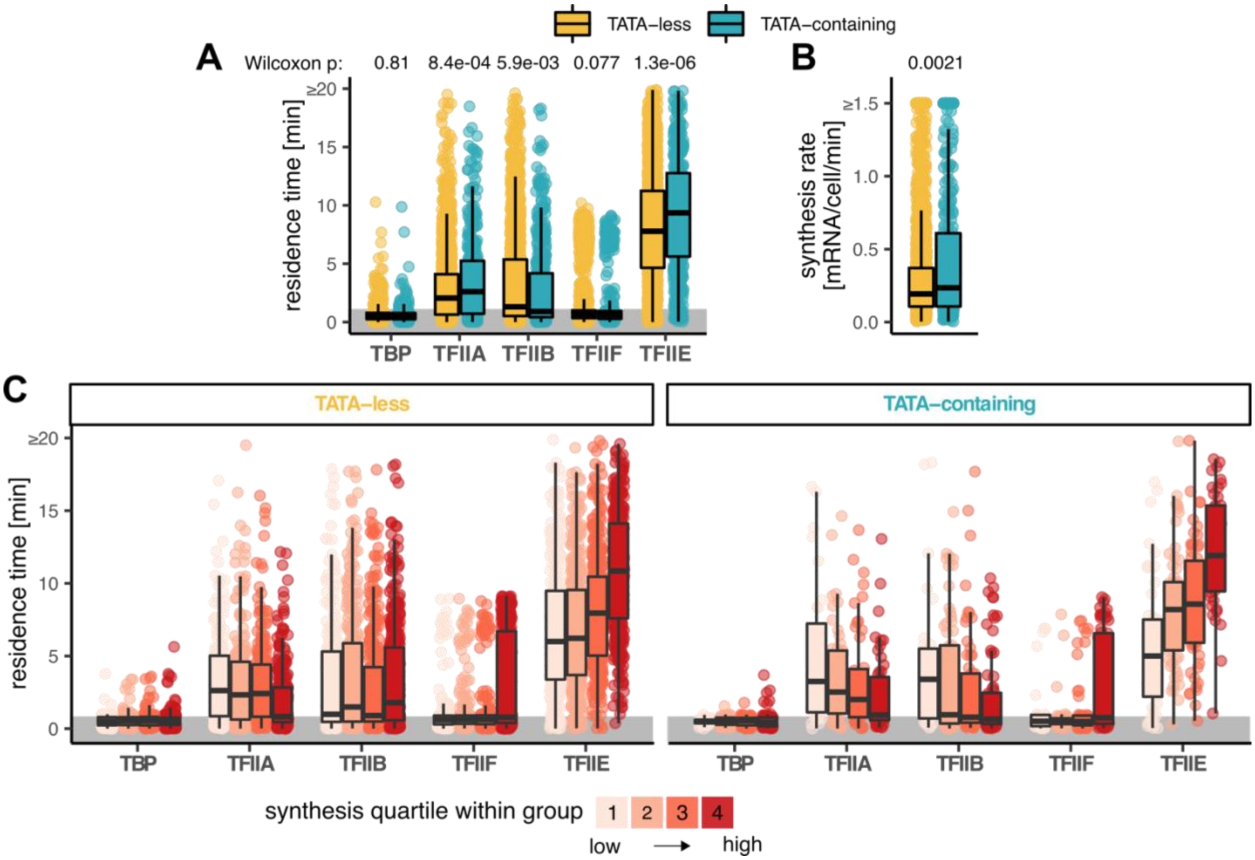

Appendix Figure S5 - Comparison of residence times between genes with or without a consensus TATA element in their promoters.

A Box plots showing comparison of residence times (y-axis) for a given GTF (x-axis) for genes without a consensus TATA element in their promoters (labeled TATA-less) and genes with a TATA-containing promoter (Basehoar *et al*, 2004).

B Box plot showing comparison of synthesis rates (y-axis) between genes with TATA-less vs. TATA-containing promoters.

C Box plots showing comparison of residence times (y-axis) for a given GTF (x-axis) across synthesis quartiles within genes with TATA-less (left panel) and TATA-containing (right panel) promoters. In the plots grey area highlights values randomly generated in this study for reliably fast sites.

Data information: (A-C) In box plots the middle line represents the median, the lower and upper hinge represent the first and third quartiles, and the whiskers represent 1.5 * interquartile range. The ≥ symbol on the y axis indicates that values higher than the indicated value were shrunk to the value shown for plotting purposes in order to eliminate outliers.

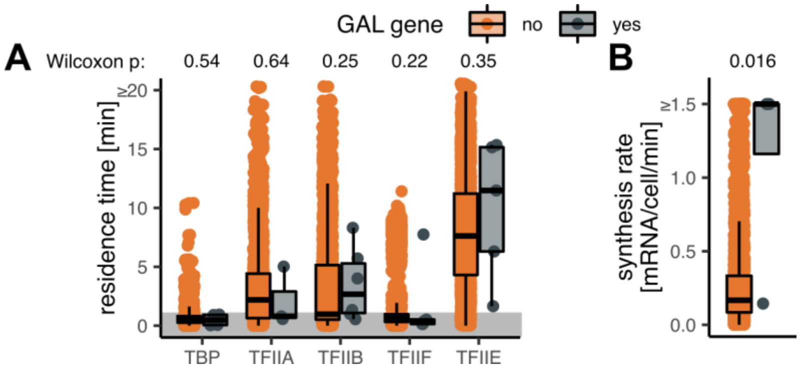

Appendix Figure S6 - Comparison of residence times between GAL genes and others.

A Box plots showing comparison of residence times (y-axis) for a given GTF (x-axis) for *GAL* genes (gray) and all other genes (orange).

B Box plot showing comparison of synthesis rates (y-axis) for *GAL* genes (gray) versus all other genes.

Data information: (A-B) The middle line in each box plot represents the median, the lower and upper edges of the rectangles represent the boundaries of the first and third quartiles, and the whiskers represent 1.5 * interquartile range. The ≥ symbols on the y axes indicate that values higher than the indicated value were shrunk for plotting purposes to the value shown to eliminate outliers.

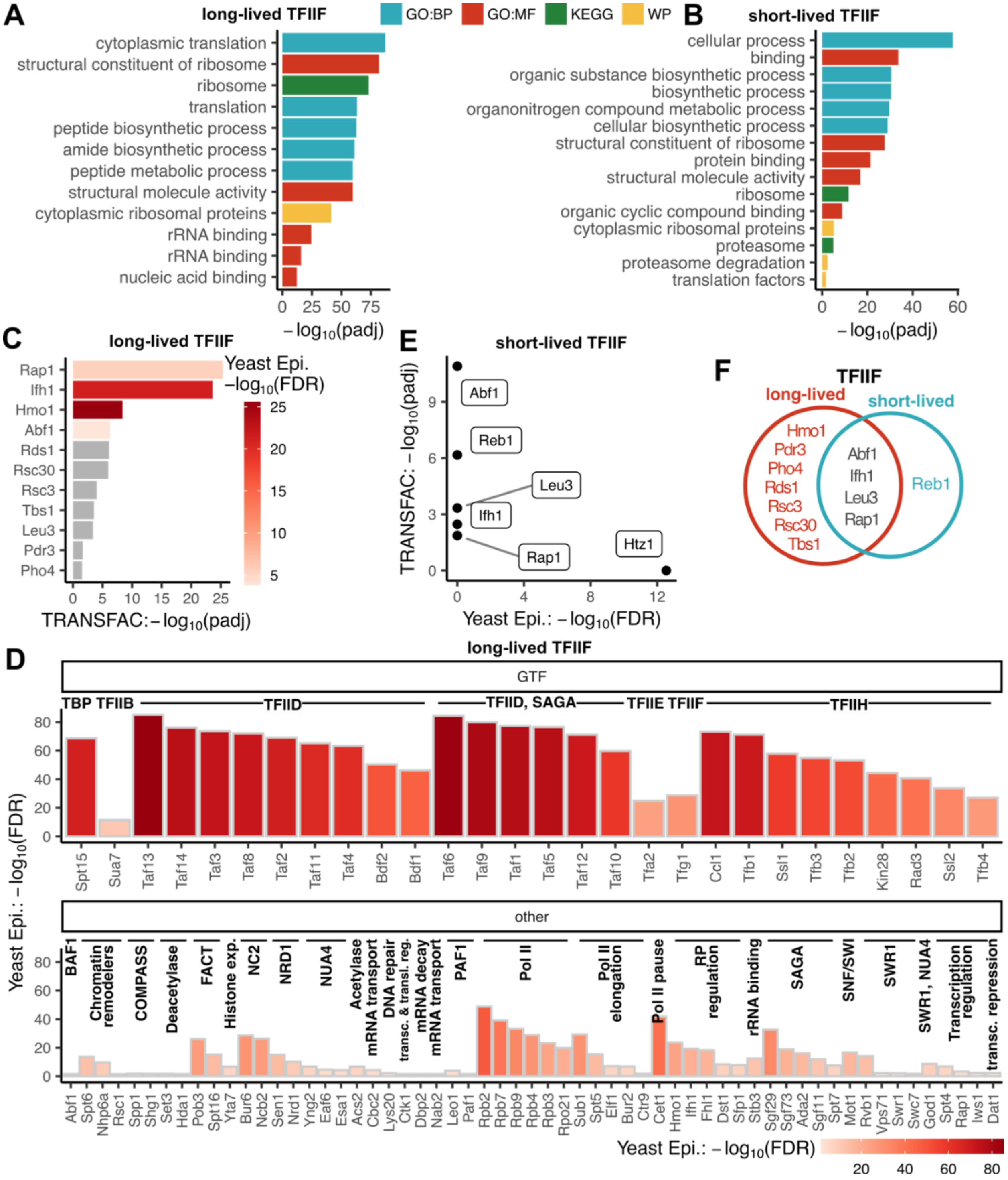

Appendix Figure S7 - Functional enrichment of long-lived and short-lived TFIIF sites.

A,B Pathway enrichment of gene targets of long-lived (A) and short-lived (B) TFIIF sites.

(C) TRANSFAC enrichment of long-lived TFIIF gene targets. TF enrichments also identified in Yeast Epigenome database are highlighted in shade of red based on Yeast Epigenome enrichment FDR-corrected p-values.

D Enrichment of long-lived TFIIF gene targets in Yeast Epigenome DBF targets. Enriched factors are separated into general transcription factors (GTF) and others and were manually classified into categories. P-values: Fisher's exact test.

E Enrichment of short-lived TFIIF gene targets withing Yeast Epigenome database on x-axis vs enrichment within TRANSFAC.

F Venn diagram showing TRANSFAC TFs enriched in long-lived vs. short-lived TFIIF sites.

Data information: As TFIIF long-lived sites were classified those with residence times ≥ 5 minutes, the rest were classified as short-lived. GO:BP GO biological process, GO:MF GO molecular function, WP WikiPatways. Significant enrichment: FDR $p < 0.05$. See Material and Methods section "Yeast DBF database (Yeast Epigenome)" for more details on Yeast Epigenome database.

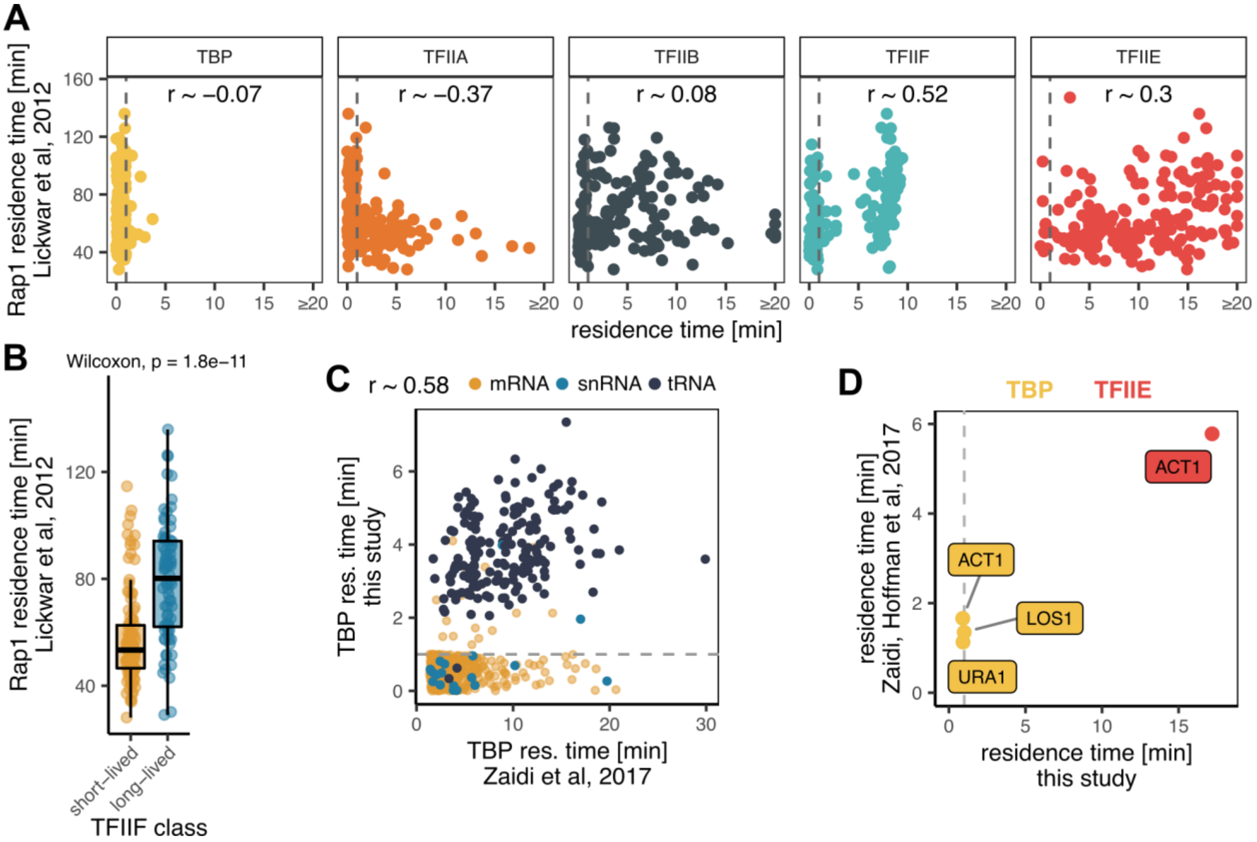

Appendix Figure S8 - Comparison to Rap1 residence times and comparison of TBP and TFIIE residence times to others.

A Comparison between residence times of GTFs from this study (x-axis) to Rap1 residence times from (Lickwar *et al*, 2012). Pearson's correlation coefficient estimates, r, are indicated in each panel. Grey dashed line separates randomly generated residence time values for reliably fact sites (<1 min).

B Box plots showing the difference in Rap1 residence times at gene targets of short-lived (<=5 minutes) vs. long-lived (>5 minutes) TFIIF sites.

C Comparison of TBP residence time estimates from this study (y-axis) to those from (Zaidi *et al*, 2017a) (x axis). Pearson's correlation coefficient estimate is indicated above the plot.

D Comparison of TBP and TFIIE residence time estimates from this study (x-axis) to those from (Zaidi *et al*, 2017b) (y-axis). In the plots grey dashed line separates values randomly generated in this study for reliably fast sites.

Data information: Symbol ≥ on the axis indicates that values higher than an indicated value were shrunk for plotting purposes to eliminate outliers. In the box plots the middle line represents the median, the lower and upper hinge represent the first and third quartiles, and the whiskers represent 1.5 * interquartile range.

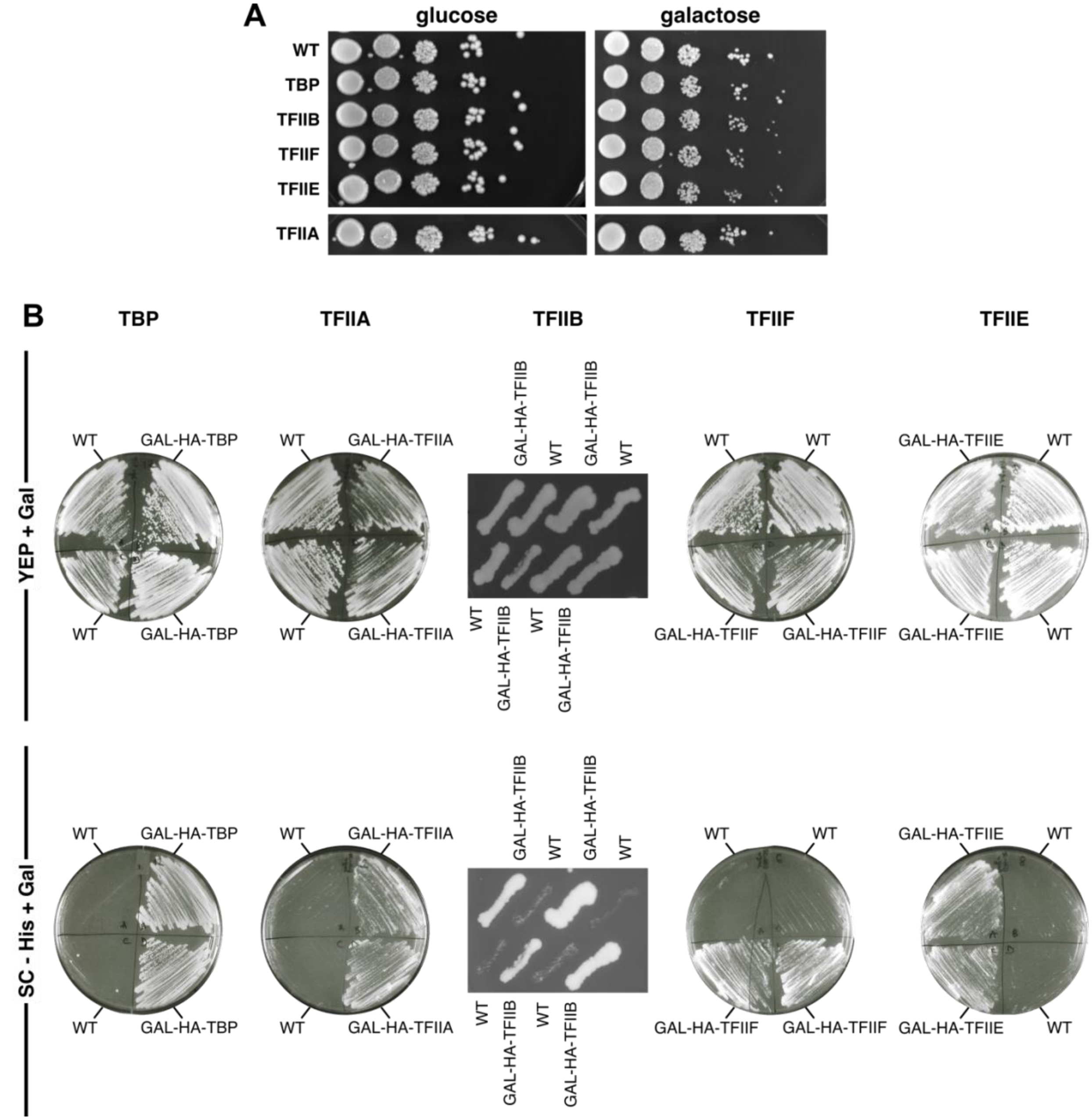

Appendix Figure S9 - Growth of competition ChIP strains.

A Ten-fold serial dilutions of diploid yeast strains carrying epitope-tagged versions of the indicated factors. In each GTF competition ChIP strain, one allele of the indicated GTF was tagged with Myc and under control of the native promoter. The other allele was tagged with HA and was under *GAL1* control. Note that for TFIIA, TFIIE, and TFIIF, which contain two subunits, one allele of the other subunit was under control of the native promoter and the other allele was untagged and under *GAL1* control. Please see Materials and Methods for additional details.

B Growth of haploid cells that contain the indicated GTF tagged with HA and under *GAL1* control as indicated. Two isolates of each strain are shown. As in (A), for TFIIA, TFIIE and TFIIF, the other subunit gene for these factors was untagged and under *GAL1* control. Growth on plates without histidine was used to score for cells carrying the HA-tagged alleles.

Data information: WT wildtype cells.

SUPPLEMENTARY TABLES

Appendix Table S1 - Quantified Western blots: HA/Myc ratios normalized to 0-1.

target	time [min]	HA/Myc ratio	HA/Myc ratio: sd
TBP	0	0.000	0.000
TBP	10	0.000	0.000
TBP	20	0.067	0.038
TBP	25	0.188	0.004
TBP	30	0.393	0.047
TBP	40	0.702	0.056
TBP	60	0.935	0.085
TBP	90	1.087	0.049
TBP	120	1.000	0.000
TFIIA	0	0.000	0.000
TFIIA	10	0.000	0.000
TFIIA	20	0.008	0.026
TFIIA	25	0.042	0.028
TFIIA	30	0.135	0.025
TFIIA	40	0.236	0.105
TFIIA	60	0.443	0.133
TFIIA	90	0.909	0.115
TFIIA	120	1.000	0.049
TFIIB	0	0.000	0.000
TFIIB	10	0.006	0.004
TFIIB	20	0.025	0.008
TFIIB	25	0.123	0.005
TFIIB	30	0.234	0.092
TFIIB	40	0.374	0.062
TFIIB	60	0.824	0.037
TFIIB	90	1.036	0.096
TFIIB	120	1.000	0.100
TFIIF	0	0.000	0.000
TFIIF	10	0.004	0.006
TFIIF	20	0.005	0.003
TFIIF	25	0.013	0.007
TFIIF	30	0.053	0.091
TFIIF	40	0.441	0.409
TFIIF	60	0.873	0.129
TFIIF	90	0.978	0.219
TFIIF	120	1.000	0.303
TFIIE	0	0.000	0.000
TFIIE	10	0.004	0.009
TFIIE	20	0.131	0.044
TFIIE	25	0.212	0.042
TFIIE	30	0.308	0.043
TFIIE	40	0.466	0.028
TFIIE	60	0.734	0.095
TFIIE	90	0.965	0.016
TFIIE	120	1.000	0.020

Appendix Table S2 - List of primers

GTF	subunit	target	forward/ reverse	primer 5'-3'
TBP	-	HisPGAL1-3HA	F	TGGACTATAAGATCGGGGGAAAGATAACACATAAGAAATAAAACgaattcgagctcgtttaaac
TBP	-	HisPGAL1-3HA	R	TCTTGTTTGCCTCTTTAAACTCCTTTAAACGTTCCTCATCGGCgcactgagcagc gtaatctg
TBP	-	N-Lox- 9xMyc	F	TTCTAGTCCAACATAAACAGGTGTATCAAGAGAAACTTTTTTAATTatgtgcaggtcgacaacccttaat
TBP	-	N-Lox-9xmyc	R	ACACTATCTTGTTTGCCTCTTTAAACTCCTTTAAACGTTCCTCATCGGCgcggccgcataggccact
TFIIA	TOA1	HisPGAL1-3HA	F	CAAATAACAAGGTGGATAAAGCCATACGGCATATTATCGgaattcgagctcgtttaaac
TFIIA	TOA1	HisPGAL1-3HA	R	ACTCTACGATAATCTCGTACACTCTGCTGGCCTCTGCATTCGAgcactgagcagcgtaatctg
TFIIA	TOA2	PGAL1-TRP	F	GTATTGCCGGGTAAGCTAATTTTACTAATAACAATATTTTagaattcgagctcgtttaac
TFIIA	TOA2	PGAL1-TRP	R	TACTTCTACGATATAATTCGTAATACCCGGGTACTGCCATtttgagatccgggtttt
TFIIA	TOA1	N-Lox-9XMyc	F	CACAAATCAAGGCCATACACAAGGGACATCGTTGGATatgtgcaggtcgacaacccttaat
TFIIA	TOA1	N-Lox-9XMyc	R	AGACTCTACGATAATCTCGTACACTCTGCTGGCCTCTGCATTCGAgcggccgcataggccact
TFIIB	-	HisPGAL1-3HA	F	AAAGTGAAGAGAATAATCATCACTTATAAAGACAACTTAATAGACGgaattcgagctcgtttaaac
TFIIB	-	HisPGAL1-3HA	R	AGGACCCCTTCTTCCTGCTCTTTTATCTATGCTCTCCCTAGTgcactgagcagcgtaatctg
TFIIB	-	N-lox-9xMyc	F	GATCCAGT GATAGAGAAG GGGAGAAGTA GATACGCAGAatgtgcaggtcgacaacccttaat
TFIIB	-	N-Lox-9xmyc	R	ATTAGGACCCCTTCTTCCTGCTCTTTTATCTATGCTCTCCCTAGTgcggccgcataggccact
TFIIE	TFA1	HisPGAL1-3HA	F	ACGCGAAACATATGAGAAATTTTTCTAACTTTTCTTCCTTCGTTAAGgaattcgagctcgtttaaac
TFIIE	TFA1	HisPGAL1-3HA	R	GAACTTTAAAAGATTTTTCACAATATCATCTATAGGTCTATCgcactgagcagcgtaatctg
TFIIE	TFA2	PGAL1-TRP	F	AAGTTCACTTACGAATCAGGGCAAAAGAGCTAATAAGAAGTGAACTagaattcgagctcgtttaac
TFIIE	TFA2	PGAL1-TRP	R	AGCGTTCAAATTAGCCAGTAGAGGGTCCCTGTTTTTACTCATtttgagatccgggtttt
TFIIE	TFA1	N-lox-9xMyc	F	ATAGAAAACTACTGGAAGCAGCTGCTGCAGTGAGTAGAGTatgtgcaggtcgacaacccttaat
TFIIE	TFA1	N-Lox-9xmyc	R	GACGAACTTTAAAAGATTTTTCACAATATCATCTATAGGTCTATCgcggccgcataggccact
TFIIF	TFG2	HisPGAL1-3HA	F	TCAGGCTTGAAATTAGAAGAATAAGACATATGCTGTTGGCTTgaatttcgagctcgtttaaac
TFIIF	TFG2	HisPGAL1-3HA	R	GGAATTATTAGAAAGTGCTGGTGCCCCTGCTGAACCACTGCTgcactgagcagcgtaatctg
TFIIF	TFG1	PGAL1-TRP	F	TGATAAAATAAAAATTATGATAGTAAGAAGTATGGATGGTagaattcgagctcgtttaac
TFIIF	TFG1	PGAL1-TRP	R	GACCTCCCCCGTTTCTACTGCCTGGTGGATTGGCGTCTGGACATtttgagatccgggtttt
TFIIF	TFG2	N-Lox-9XMYC	F	CAATATTAAGAGTAAAATAGAAGACCAACTTAACACCatgtgcaggtcgacaacccttaa
TFIIF	TFG2	N-Lox-9XMYC	R	GGAATTATTAGAAAGTGCTGGTGCCCCTGCTGAACCACTGCTgcggccgcataggccact

Appendix Table S3 - Hill model fits to HA/Myc Western blot data for turnover model

GTF	XP value	XP standard error	XP t-statistic	XP p-value	t1/2ind value [min]	t1/2ind standard error [min]	t1/2ind t-statistic	t1/2ind p-value	Hill coeff n
TBP	1.06	0.02	44.76	7.30E-10	34.74	0.91	38.01	2.30E-09	4
TFIIA	1.21	0.06	20.07	1.91E-07	67.42	3.48	19.35	2.46E-07	3
TFIIB	1.62	0.04	39.19	1.83E-09	44.25	1.32	33.48	5.50E-09	4
TFIIF	2.79	0.04	68.65	3.66E-11	41.71	0.56	73.93	2.18E-11	7
TFIIE	1.03	0.02	46.38	5.66E-10	41.54	1.16	35.69	3.52E-09	3

Appendix Table S4 - Accurate initial estimates of residence times for turnover model fits:

$$\boldsymbol{t^0_{1/2}[min] = b_0 + b_1(t_{1/2CC} - t_{1/2ind}) + b_2(t_{1/2CC} - t_{1/2ind})^2}$$

GTF	b0	b1	b2	b0 for (t1/2CC - t1/2ind) ≤ 4 [min]	b1 for (t1/2CC - t1/2ind) > 4 [min]
TBP	0.082	0.608	0		
TFIIA	0.201	0.607	0		
TFIIB	0.454	0.575	0		
TFIIF*	0.205	0.238	0.164	3.61	0.266
TFIIE	-0.037	0.629	0		

* TFIIF $t_{1/2}$ vs ($t_{1/2CC}$ - $t_{1/2ind}$) plots displayed a quadratic relationship for ($t_{1/2CC}$ - $t_{1/2ind}$) ≤ 4 min followed by a linear relationship for ($t_{1/2CC}$ - $t_{1/2ind}$) > 4 min

SUPPLEMENTARY REFERENCES

Basehoar AD, Zanton SJ & Pugh BF (2004) Identification and Distinct Regulation of Yeast TATA Box-Containing Genes. *Cell* 116: 699–709

García-Martínez J, Aranda A & Pérez-Ortín JE (2004) Genomic Run-On Evaluates Transcription Rates for All Yeast Genes and Identifies Gene Regulatory Mechanisms. *Mol Cell* 15: 303–313

Lickwar CR, Mueller F, Hanlon SE, McNally JG & Lieb JD (2012) Genome-wide protein–DNA binding dynamics suggest a molecular clutch for transcription factor function. *Nature 2012 484:7393* 484: 251–255

Zaidi HA, Auble DT & Bekiranov S (2017a) RNA synthesis is associated with multiple TBP-chromatin binding events. *Scientific Reports 2017 7:1* 7: 1–12

Zaidi HA, Hoffman EA, Shetty SJ, Bekiranov S & Auble DT (2017b) Second-generation method for analysis of chromatin binding with formaldehyde–cross-linking kinetics. *Journal of Biological Chemistry* 292: 19338–19355
