# Supplementary figures and images for: Genome-scale chromatin binding dynamics of RNA Polymerase II general transcription machinery components
Source: EMBO J. 2024 Apr 2;43(9):1799–821. doi: 10.1038/s44318-024-00089-2 (PMC11066129; doi:10.1038/s44318-024-00089-2)

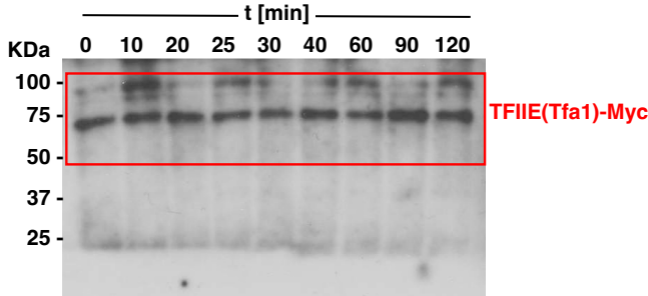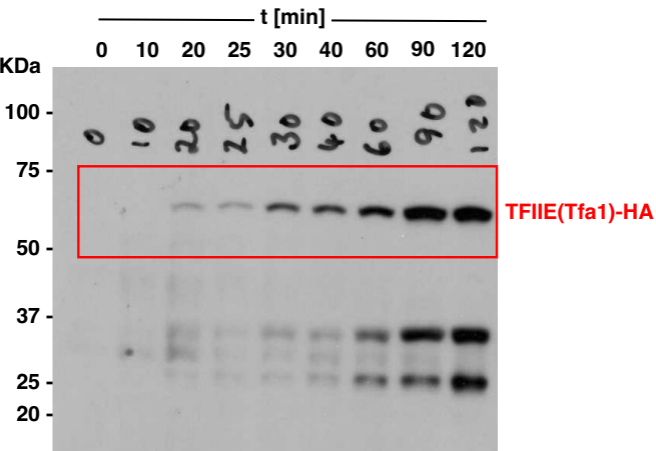

Supplement: Supplementary file 5 — Source data Fig. 2 [file 44318_2024_89_MOESM5_ESM.zip › Figure 2/2A/TFIIE_WesternBlot.pdf]

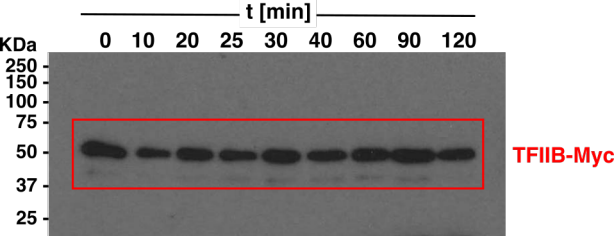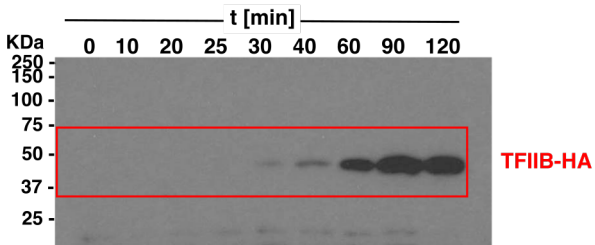

Supplement: Supplementary file 7 — Expanded View and Appendix Source Data [file 44318_2024_89_MOESM7_ESM.zip › EMBOJ-2023-115213_SourceDataForExpandedViewAndAppendix/Figure EV1/EV1C/TFIIB_WesternBlot.pdf]

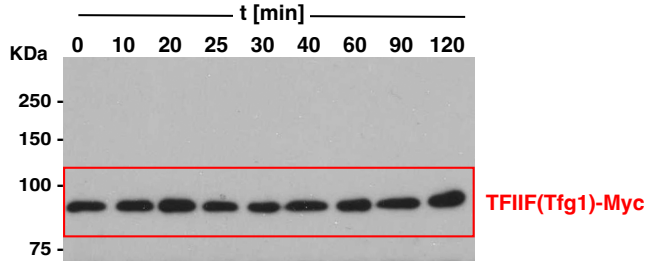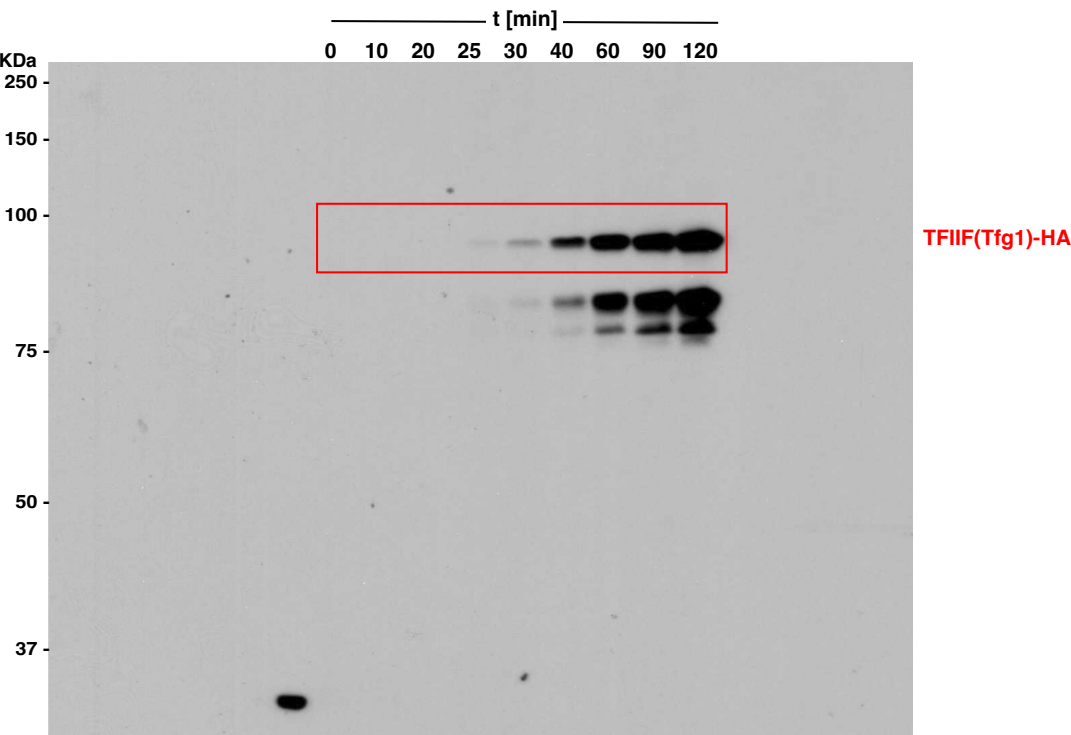

Supplement: Supplementary file 7 — Expanded View and Appendix Source Data [file 44318_2024_89_MOESM7_ESM.zip › EMBOJ-2023-115213_SourceDataForExpandedViewAndAppendix/Figure EV1/EV1D/TFIIF_WesternBlot.pdf]

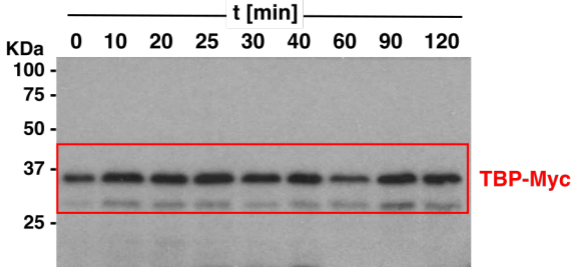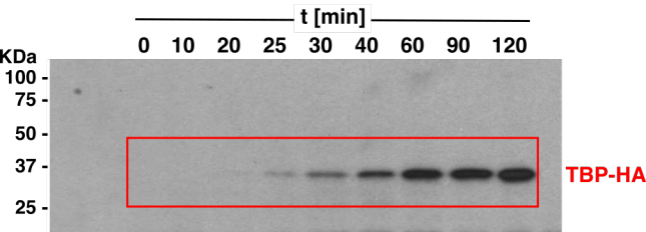

Supplement: Supplementary file 7 — Expanded View and Appendix Source Data [file 44318_2024_89_MOESM7_ESM.zip › EMBOJ-2023-115213_SourceDataForExpandedViewAndAppendix/Figure EV1/EV1A/TBP_WesternBlot.pdf]
