# Supplementary material for: Genome-scale chromatin binding dynamics of RNA Polymerase II general transcription machinery components
Source: EMBO J. 2024 Apr 2;43(9):1799–821. doi: 10.1038/s44318-024-00089-2 (PMC11066129; doi:10.1038/s44318-024-00089-2)
Supplement: Supplementary file 7 — Expanded View and Appendix Source Data [file TFIIA_WesternBlot.pdf › EMBOJ-2023-115213_SourceDataForExpandedViewAndAppendix/Figure EV1/EV1B/TFIIA_WesternBlot.pdf]

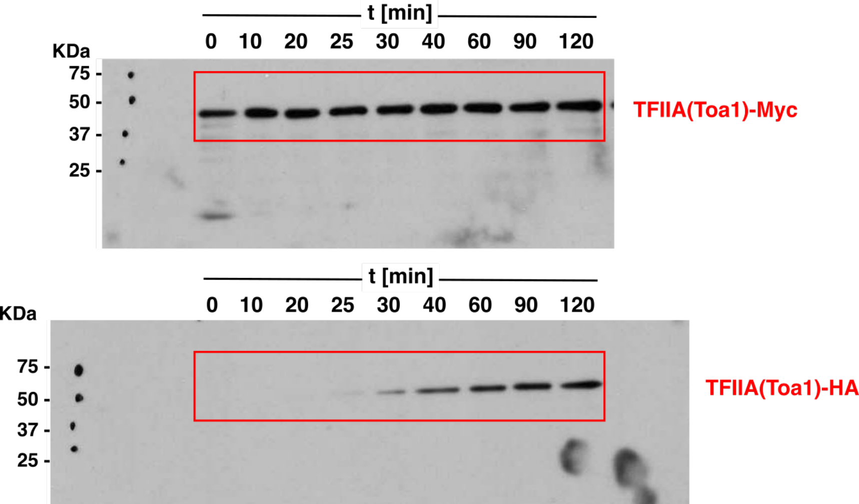
t [min]
0 10 20 25 30 40 60 90 120
KDa
75
50
37
25
TFIIA(Toa1)-Myc
t [min]
0 10 20 25 30 40 60 90 120
KDa
75
50
37
25
TFIIA(Toa1)-HA
