# Supplementary material for: Genome-scale chromatin binding dynamics of RNA Polymerase II general transcription machinery components
Source: EMBO J. 2024 Apr 2;43(9):1799–821. doi: 10.1038/s44318-024-00089-2 (PMC11066129; doi:10.1038/s44318-024-00089-2)
Supplement: Supplementary file 9 — Expanded View Figures [file 44318_2024_89_MOESM9_ESM.pdf]

Expanded View Figures

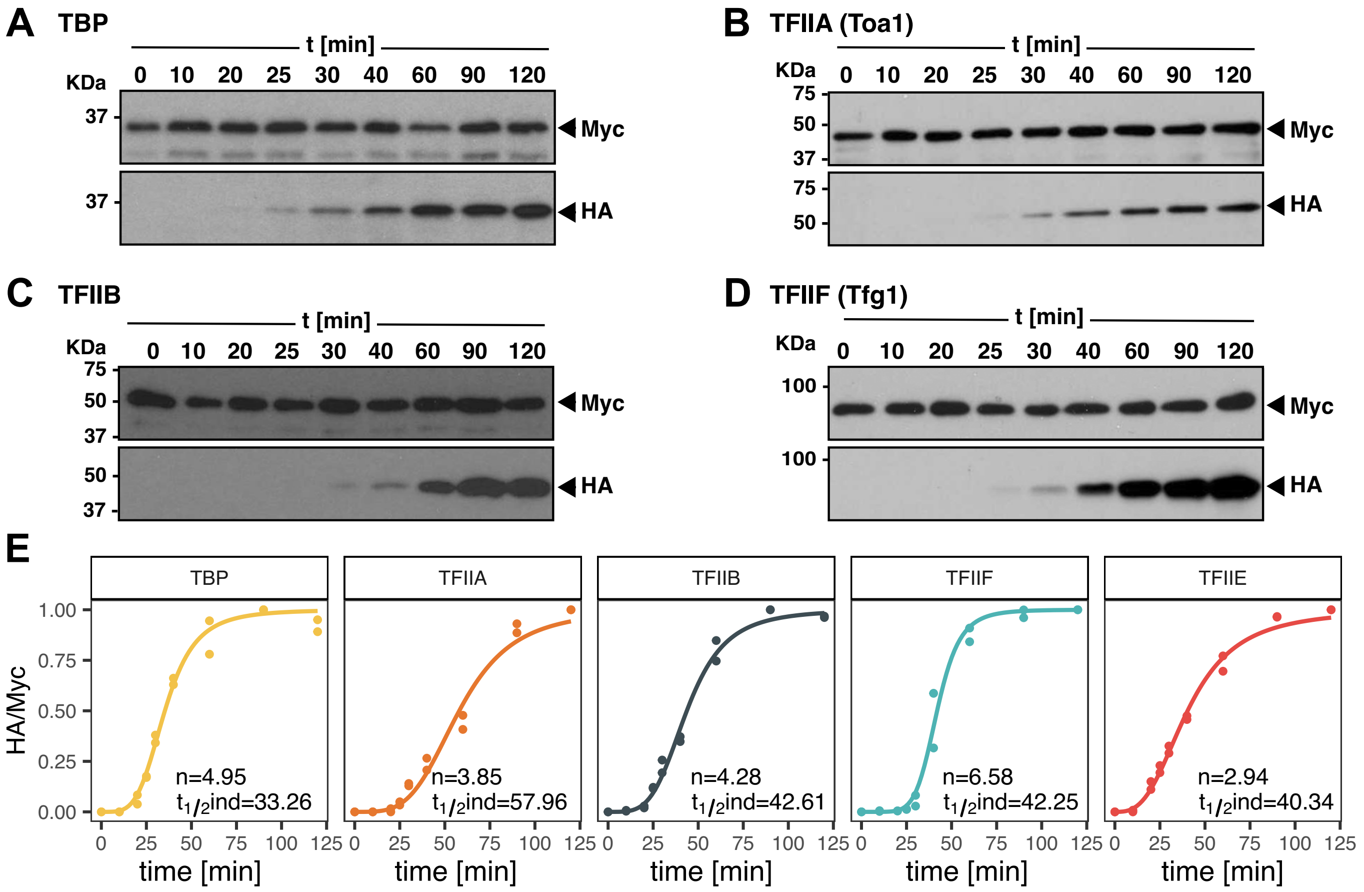

Figure EV1. Protein induction.

(**A–D**) Western blots of (**A**) TBP, (**B**) TFIIA (Toa1), (**C**) TFIIB, and (**D**) TFIIF (Tfg1) over the indicated time course. Myc tag indicates proteins made from genes expressed under the control of their endogenous promoters, and the HA tag measures the level of the competitor expressed under galactose control. (**E**) Normalized HA/Myc ratios quantified from Western blots ($n = 2$ biological replicates) with Hill fits. Hill fit parameters are shown in the bottom right corner of each panel, n Hill coefficients, $t_{1/2}$ind half-time of HA-tagged protein induction. Source data are available online for this figure.

 © The Author(s)

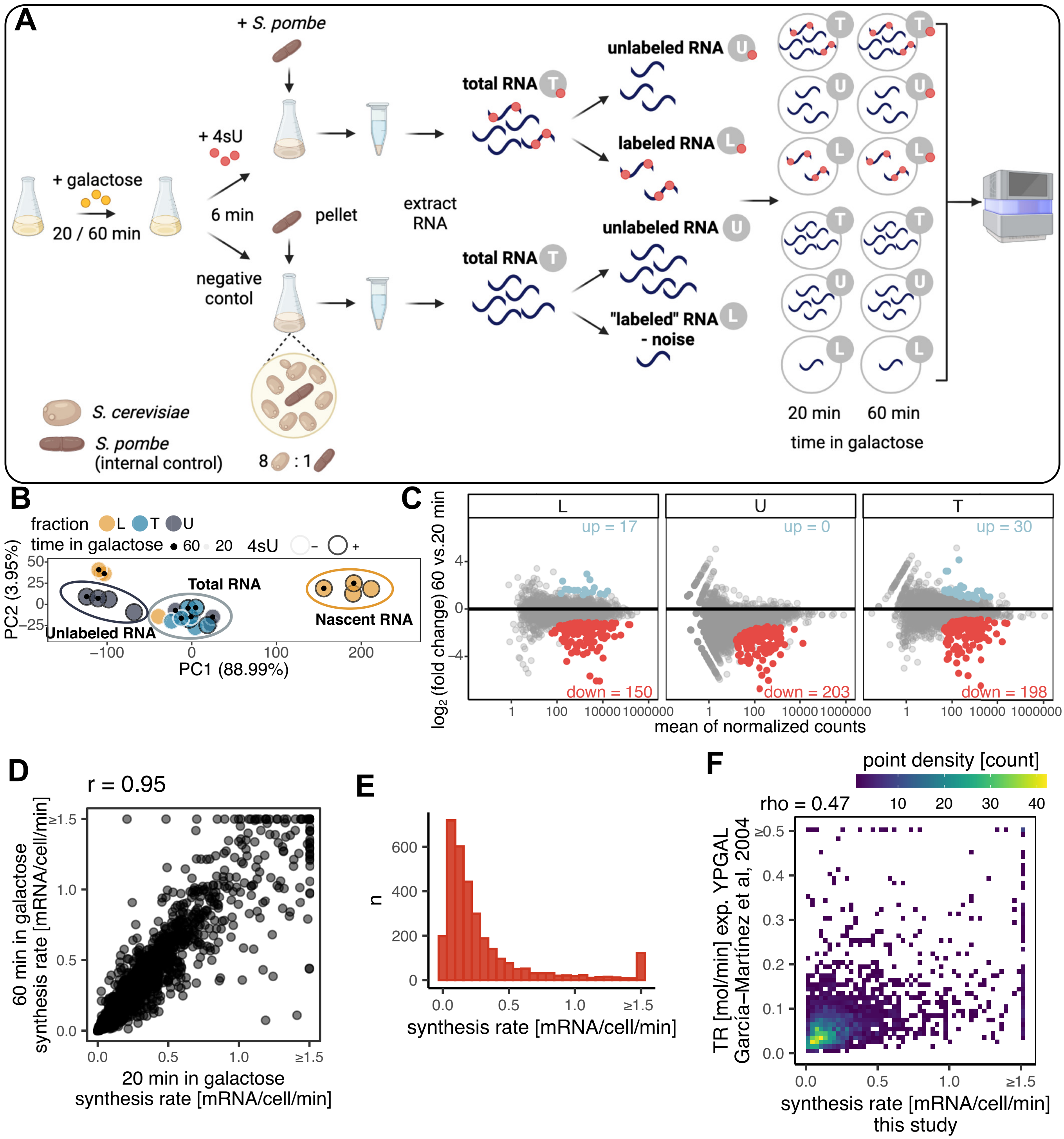
A
+ S. pombe
+ galactose
20 / 60 min
+ 4sU
6 min
negative contol
pellet
extract RNA
total RNA
unlabeled RNA
labeled RNA
"labeled" RNA - noise
S. cerevisiae
S. pombe (internal control)
8 : 1
20 min
60 min
time in galactose
B
fraction L T U
time in galactose 60 20 4sU – +
Total RNA
Unlabeled RNA
Nascent RNA
PC1 (88.99%)
PC2 (3.95%)
C
L
U
T
up = 17
up = 0
up = 30
down = 150
down = 203
down = 198
log2 (fold change) 60 vs.20 min
mean of normalized counts
D
r = 0.95
60 min in galactose synthesis rate [mRNA/cell/min]
20 min in galactose synthesis rate [mRNA/cell/min]
E
n
synthesis rate [mRNA/cell/min]
F
point density [count]
rho = 0.47
TR [mol/min] exp. YPGAL García-Martinez et al, 2004
synthesis rate [mRNA/cell/min] this study

◀ **Figure EV2. Synthesis rate estimation with dynamic transcriptome analysis (DTA).**

(**A**) Schematic overview of the DTA method as adapted in this study. (**B**) Principal component analysis (PCA) plot showing the first two principal components (PCs) calculated from normalized read coverage signal from all samples generated in this study. Highlighted are clusters of samples representing nascent RNA (L fraction after 4-sU addition), total RNA (T fraction after 4-sU addition as well as from negative control, along with U fraction from negative control), and unlabeled RNA (U fraction after 4-sU addition). Percentages within the axis labels indicate the percentage of variance explained by a given PC. (**C**) MA plot showing differentially expressed genes between samples grown for 60 vs. 20 min in galactose. Each point represents a gene, the x-axis indicates the size of a given gene in terms of the mean number of reads after normalization mapped to the gene, and the y-axis shows $\log_2$ of fold change between the two conditions. Highlighted are significantly misregulated genes, blue: upregulated at 60 min, red: downregulated at 60 min compared to the 20-min time point, significance threshold: FDR-corrected *p* value (*p*adj) <0.05. (**D**) Comparison of synthesis rates (in mRNA per cell per minute) estimated from samples grown for 20 min in galactose (x-axis) vs. 60 min in galactose (y-axis). Pearson's correlation coefficient can be found above the plot. (**E**) Histogram showing the distribution of synthesis rates (in mRNA per cell per minute) estimated jointly from samples grown for 20 and 60 min in galactose. Synthesis rates higher than 1.5 were combined into one bar to eliminate long tails. (**F**) Comparison of synthesis rates generated in this study (x-axis) to those generated by García-Martínez et al, 2004 (García-Martínez et al, 2004). Spearman's correlation coefficient is mentioned above in the plot. The plot is color-coded based on point density in each area. Symbol ≥ on the axis indicates that values higher than an indicated value were shrunk for plotting purposes to eliminate outliers.

 © The Author(s)

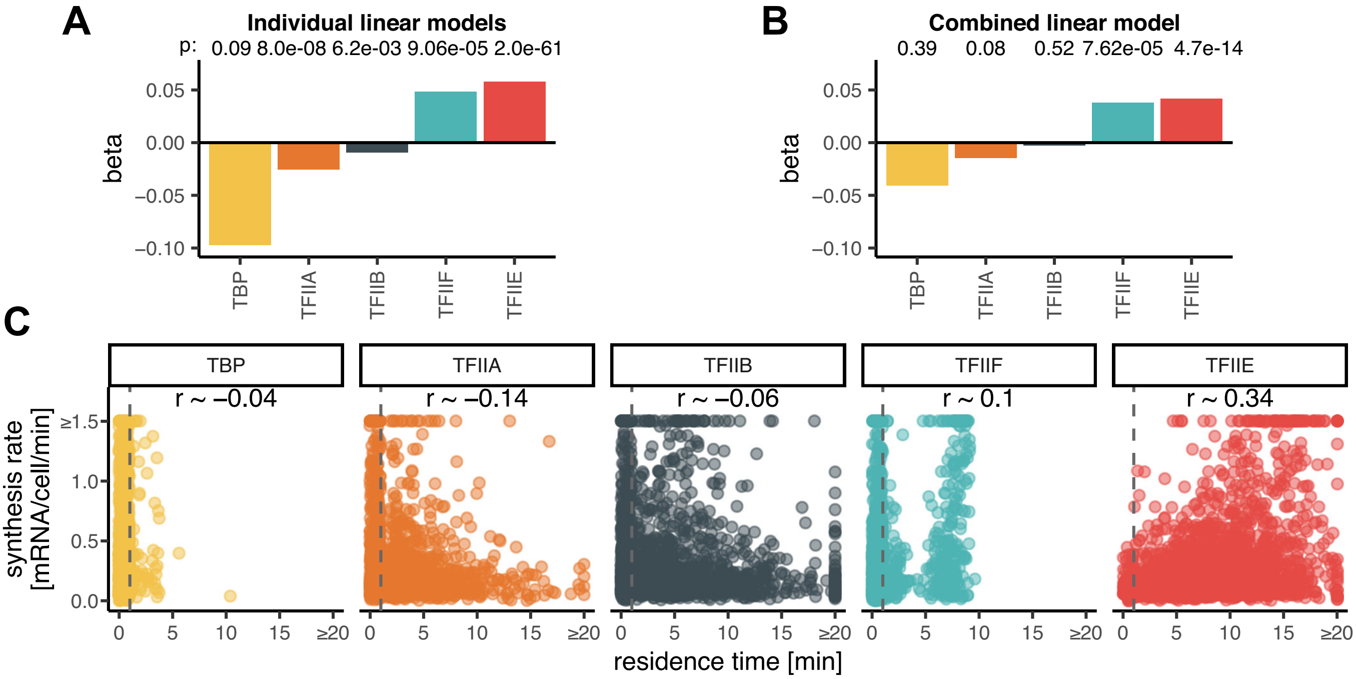

Figure EV3. Comparison between residence times and synthesis rates.

(**A**) Bar plot showing β (beta) coefficients for linear models built between synthesis rates and residence times of an indicated GTF (synthesis rate ~ β*res. $time_{GTF} + \alpha$). (**B**) Analogous to (**A**), showing coefficient from a linear model combining all factors (synthesis rate ~ β_{TBP}*res. $time_{TBP} + \beta_{TFIIA}$*res. $Time_{TFIIA} + \beta_{TFIIB}$*res. $Time_{TFIIB} + \beta_{TFIIF}$*res. $Time_{TFIIF} + \beta_{TFIIE}$*res. $Time_{TFIIE} + \alpha$). (**C**) Relationship between residence times (x-axis) and synthesis rates (y-axis) for GTFs as indicated. Pearson's correlation coefficient estimates, r, are indicated in each panel. Symbol ≥ on the axis indicates that values higher than an indicated value were shrunk for plotting purposes to eliminate outliers. In the plots, gray dashed line separates values randomly generated in this study for reliably fast sites.

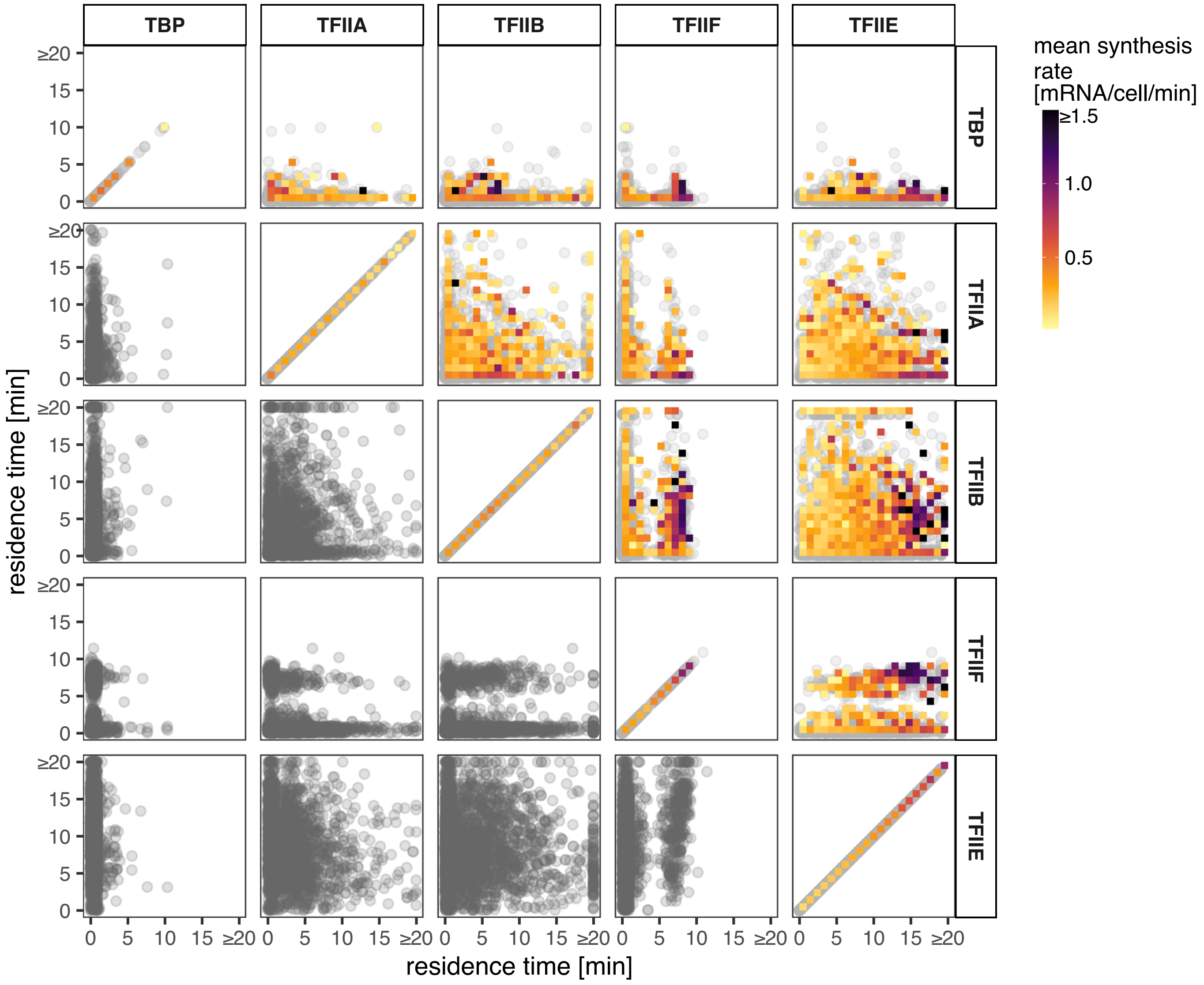

Figure EV4. Relationships among GTF residence times and to mRNA synthesis rates.

Each panel shows a comparison of residence times of pairs of GTFs as indicated in the panel titles. Each point is a shared gene target. The color map shows the mean synthesis rates of the genes under the given area. Source data are available online for this figure.

 © The Author(s)

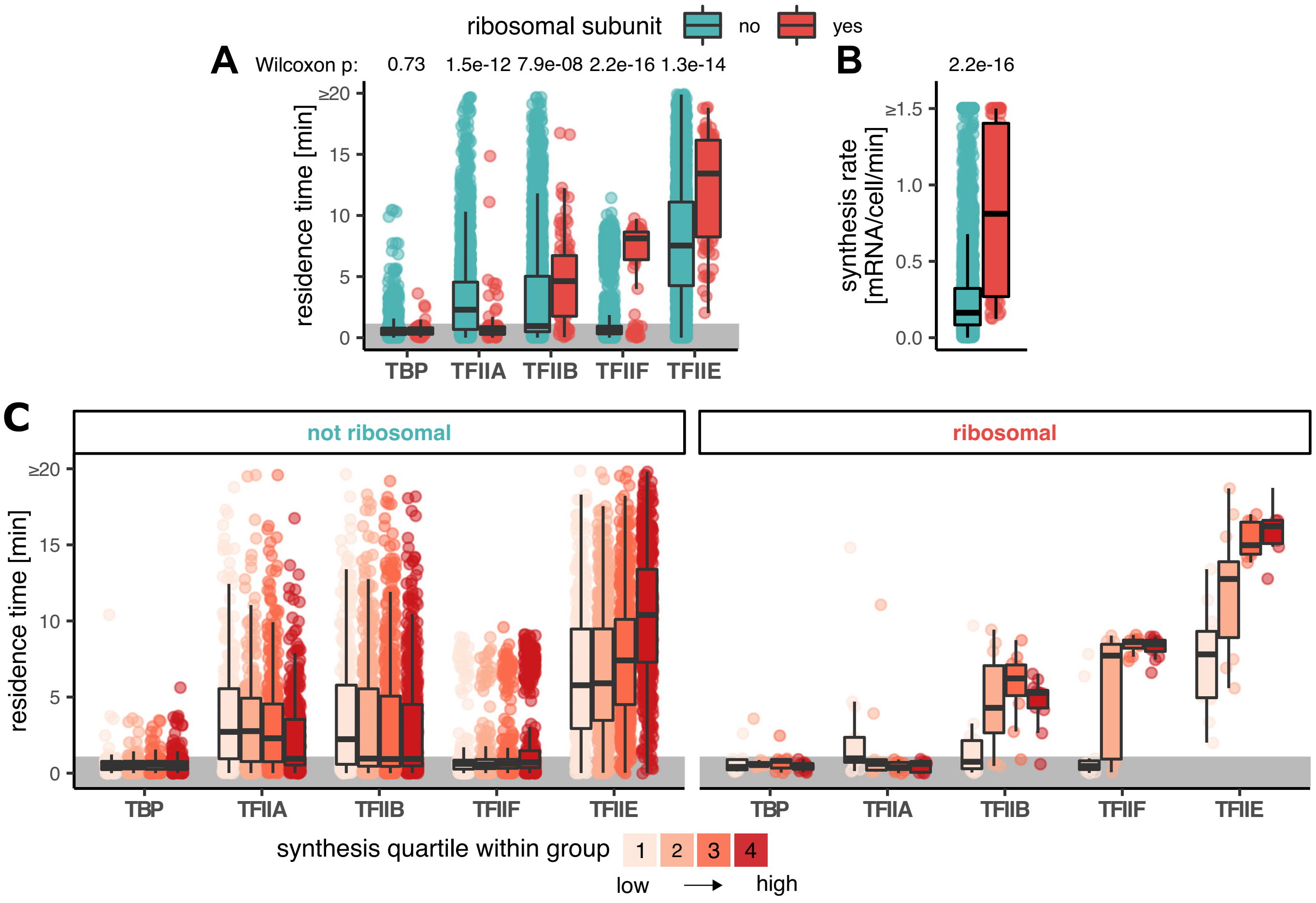

Figure EV5. Comparison of residence times between genes coding for ribosomal subunits and others.

(**A**) Box plots showing the comparison of residence times (y-axis) for a given GTF (x-axis) for genes coding for ribosomal subunits (red) and other genes (green). The number of observations (*n*): not ribosomal/ribosomal TBP- 2810/87; TFIIA- 2418/76; TFIIB- 3420/93; TFIIF- 2776/88; TFIIE- 3723/86. (**B**) Box plot showing the comparison of synthesis rates (y-axis) between genes coding for ribosomal subunits and other genes. Wilcoxon *p* value is indicated. Number of observations (*n*): not ribosomal- 3169, ribosomal- 57. (**C**) Box plots showing the comparison of residence times (y-axis) for a given GTF (x-axis) across synthesis quartiles within genes coding for ribosomal subunits and other genes. In the plots, the gray area highlights values randomly generated in this study for reliably fast sites. Symbol ≥ on the axis indicates that values higher than an indicated value were shrunk for plotting purposes to eliminate outliers. The number of observations (*n*): not ribosomal/ribosomal TBP- Q1: 209/11, Q2: 320/10, Q3: 414/11, Q4: 583/14; TFIIA- Q1: 231/13, Q2: 316/9, Q3: 382/13, Q4: 409/9; TFIIB Q1: 293/14, Q2: 475/12, Q3: 569/11, Q4: 589/12; TFIIF- Q1: 245/11, Q2: 355/12, Q3: 419/12, Q4: 494/12; TFIIE- Q1: 403/13, Q2: 577/12, Q3: 598/10, Q4:540/10. Data information: (**A**–**C**) In boxplots, the middle line represents the median, the lower and upper hinges represent the first and third quartiles, and the whiskers represent the 1.5 * interquartile range.

© The Author(s)
